# Data augmentation via diffusion model to enhance AI fairness

**DOI:** 10.3389/frai.2025.1530397

**Published:** 2025-03-19

**Authors:** Christina Hastings Blow, Lijun Qian, Camille Gibson, Pamela Obiomon, Xishuang Dong

**Affiliations:** ^1^Prairie View A&M University, Electrical and Computer Engineering, Texas A&M University System, Prairie View, TX, United States; ^2^Texas Juvenile Crime Prevention Center, Prairie View A&M University, College of Juvenile Justice, Texas A&M University System, Prairie View, TX, United States

**Keywords:** generative AI, AI fairness, AIF360, reweighting samples, COMPAS dataset, adult income dataset

## Abstract

**Introduction:**

AI fairness seeks to improve the transparency and explainability of AI systems by ensuring that their outcomes genuinely reflect the best interests of users. Data augmentation, which involves generating synthetic data from existing datasets, has gained significant attention as a solution to data scarcity. In particular, diffusion models have become a powerful technique for generating synthetic data, especially in fields like computer vision.

**Methods:**

This paper explores the potential of diffusion models to generate synthetic tabular data to improve AI fairness. The Tabular Denoising Diffusion Probabilistic Model (Tab-DDPM), a diffusion model adaptable to any tabular dataset and capable of handling various feature types, was utilized with different amounts of generated data for data augmentation. Additionally, reweighting samples from AIF360 was employed to further enhance AI fairness. Five traditional machine learning models—Decision Tree (DT), Gaussian Naive Bayes (GNB), K-Nearest Neighbors (KNN), Logistic Regression (LR), and Random Forest (RF)—were used to validate the proposed approach.

**Results and discussion:**

Experimental results demonstrate that the synthetic data generated by Tab-DDPM improves fairness in binary classification.

## 1 Introduction

In our rapidly evolving society, artificial intelligence (AI) has become a ubiquitous presence, influencing everyday activities like online banking and digital assistants. However, how can we ensure the fairness of AI-generated outcomes? AI fairness seeks to enhance the transparency and explainability of AI systems (Li et al., 2023a). It scrutinizes the results to determine if they genuinely consider the users' best interests. Additionally, guidelines are being established to ensure the safety of both corporations and consumers. Various fairness tools have been developed to address the growing need to mitigate AI biases (Richardson and Gilbert, 2021). For example, AIF360 (Bellamy et al., 2019) offers a comprehensive set of fairness metrics for datasets and models, explanations for these metrics, and algorithms to reduce bias in datasets and models concerning protected attributes such as sex and race.

Data augmentation (Ding et al., 2019) aims to generate synthetic data from existing datasets to enlarge the training data to enhance the machine learning performance (Bansal et al., 2022). This technique increases both the quantity and variety of data available for training and testing models, eliminating the need for new data collection. Data augmentation can be achieved by either learning a generator, such as through GAN networks (Alqahtani et al., 2021), to create data from scratch, or by learning a set of transformations to apply to existing training set samples (Cubuk et al., 2019). Both approaches enhance the performance of deep learning models by providing a more diverse and abundant dataset.

In recent years, diffusion models (Yang et al., 2023) have emerged as a powerful technique for generating synthetic data to address data scarcity. For example, Villaizán-Vallelado et al. (2024) proposed a diffusion model for generating synthetic tabular data with three key enhancements: a conditioning attention mechanism, an encoder-decoder transformer as the denoising network, and dynamic masking. Nguyen et al. (2024) introduced a novel method for generating pixel-level semantic segmentation labels using the text-to-image generative model Stable Diffusion (SD), which incorporates uncertainty regions into the segmentation to account for imperfections in the pseudo-labels. Additionally, Hu et al. (2024) developed a novel diffusion GNN model called Syngand, capable of generating ligand and pharmacokinetic data end-to-end, providing a methodology for sampling pharmacokinetic data for existing ligands using this model.

In the context of tabular data augmentation (Cui et al., 2024), GANs (Goodfellow et al., 2020), and Variational Autoencoders (VAEs) (Kingma et al., 2019) offer distinct methodologies. GANs excel at capturing complex data distributions, making them highly effective for generating realistic tabular data. However, their training process is often unstable due to the adversarial setup, requiring meticulous tuning. In contrast, VAEs provide stable training through their probabilistic framework and are adept at learning latent representations, enabling data interpolation and exploration. Despite these advantages, VAEs tend to generate less sharp or realistic data compared to GANs, and balancing reconstruction loss with regularization remains a challenge. Diffusion models, compared to GANs and VAEs, present a promising alternative. They leverage a robust theoretical foundation for stable training to deliver high-quality generated data, addressing some of the limitations of GANs and VAEs. However, this advantage comes at the cost of higher computational requirements. Therefore, this study utilized diffusion model-based methods for tabular data augmentation to investigate enhancements in AI fairness.

This paper aims to investigate whether diffusion models can generate synthetic data to enhance AI fairness as well as machine learning performance. Tabular Denoising Diffusion Probabilistic Model (TabDDPM) (Kotelnikov et al., 2023) is a diffusion model that can be universally applied to any tabular dataset, handling all feature types. It uses multinomial diffusion for categorical and binary features, and Gaussian diffusion for numerical ones. Tab-DDPM effectively manages mixed data types and consistently generates high-quality synthetic data. It is used to conduct different increments of generated data samples, specifically 20,000, 100,000, and 150,000 samples. To further mitigate bias, reweighting samples was employed to recalibrate the data. This involves adjusting the significance or contribution of individual samples within the training dataset, making it possible to remove discrimination concerning sensitive attributes without altering existing labels (Calders et al., 2009). We used techniques from AIF360 (Bellamy et al., 2019) to determine these weights, based on the frequency counts associated with the sensitive attribute. To validate the proposed method, five traditional machine learning models were applied: Decision Tree (DT), Gaussian Naive Bayes (GNB), K Nearest Neighbor (KNN), Logistic Regression (LR), and Random Forest (RF). Experimental results indicate that the synthetic data generated by Tab-DDPM enhances the fairness of binary classification. For instance, both RF performance in binary classification and fairness evaluated by five evaluation metrics has been improved when enlarging the training data with the generated data.

The contributions of this paper can be summarized as:

1. Introduction of generative AI techniques for generating synthetic data to enhance AI fairness as well as machine learning performance.
2. Extensive experiments demonstrating that the fairness of different machine learning models can be improved with respect to various protected attributes.

## 2 Methodology

This paper aims to examine the effectiveness of Tab-DDPM and sample reweighting in enhancing the fairness of traditional machine learning algorithms on classification tasks, focusing on two key AI techniques: diffusion models and sample reweighting.

### 2.1 TabDDPM

**Figure 1:**
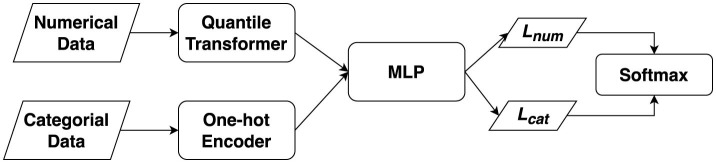
Tab-DDPM framework.

TabDDPM (Kotelnikov et al., 2023) is a generative model for tabular data, an area of active research. Tabular datasets are often limited in size due to privacy concerns during data collection. Generative AI, like Tab-DDPM, can create new synthetic data without these privacy issues. It is a newly developed model capable of effectively generating new data from tabular datasets. In detail, the DDPM process consists of three main components: the forward process, the backward process, and the sampling procedure (Chang et al., 2023). The forward process adds noise to the training data. The reverse process trains denoising networks to iteratively remove noise, differing from generative adversarial networks (GANs) by removing noise over two timesteps instead of one. The sampling procedure uses the optimized denoising network to generate novel data. It uses a Gaussian diffusion model for numerical data and a Multinomial diffusion model for categorical and binary features. Hyperparameters play a crucial role in TabDDPM, significantly influencing the model's effectiveness. The general framework of TabDDPM is shown as Figure 1.

The numerical and categorical data were represented through two branches: quantile transformer for numerical data and one-hot encoding for categorical data. These new data representations were then fed into a DDPM process utilizing multilayer perceptrons (MLP) to minimize two types of losses *L*_*num*_ and *L*_*cat*_ using softmax function.

### 2.2 Reweighting samples

Reweighting samples is a preprocessing technique that adjusts the significance or contribution of samples within a training dataset. Weights are strategically assigned making it possible to render datasets free from discrimination pertaining to sensitive attributes without altering existing labels. One such approach is by based on the frequency counts associated with the sensitive attribute (Calders et al., 2009).

This paper utilized the reweighting sample technique from the AIF360 toolbox for reweighting during the preprocessing phase. The contribution of the reweighting process comprises the training dataset with generated data of different increments with these samples containing attributes (including a sensitive attribute) and labels along with the specification of the sensitive attribute. The result being a transformed dataset where sample weights are adjusted for the sensitive attributes, mitigating potential classification bias. Throughout the reweighting process, an analysis of the allocation of the sensitive attributes within various groups is conducted. This analysis informs the calculation of reweighting coefficients, which, in turn, amends the sample weights to encourage a more uniform distribution across groups (Blow et al., 2024). For instance, given a sensitive (protected) attribute, the privileged group of these samples includes the samples with the positive sensitive attribute while the unprivileged group of samples includes the samples with the negative sensitive attribute.

### 2.3 Proposed method

**Figure 2:**
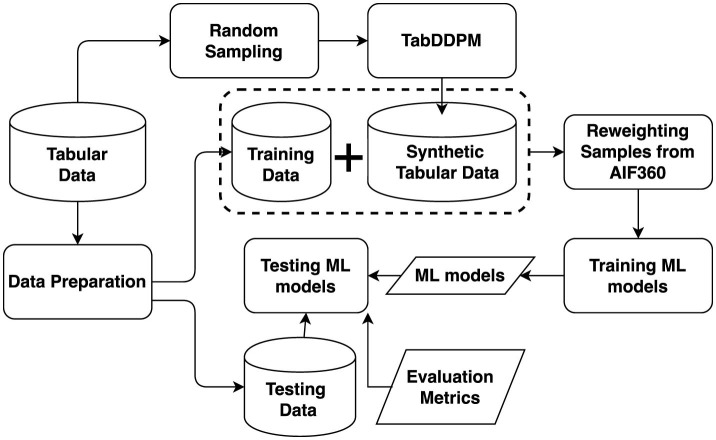
Flow of the proposed method.

The flow of the proposed method is depicted in Figure 2. The process begins with the random sampling of data, which serves as input to TabDDPM to generate synthetic tabular data. This synthetic data is then combined with the original training data to create a comprehensive dataset for training the ML model. In addition, reweighting samples from AIF360 is employ to adjust weights of different categories of samples to enhance fairness. Finally, the trained ML model is evaluated using test data, with performance assessed through multiple evaluation metrics, including various fairness metrics.

TabDDPM processes numerical and categorical features using Gaussian diffusion and Multinomial diffusion, respectively. For instance, a tabular data sample *x* = < *x*_*nu*_*m*__1__, ..., *x*_*nu*_*m*__*N*__, *x*_*c*_1__, ..., *x*_*c*_*N*__> consists of *num*_*N*_ numerical features and *c*_*N*_ categorical features. Specifically, TabDDPM applies a Gaussian quantile transformation to process each categorical feature through a separate forward diffusion process, where noise components for all features are sampled independently. The reverse diffusion step in TabDDPM is executed by a multi-layer neural network, which produces an output with the same dimensionality as the input.

Reweighting samples involves adjusting the weights of four categories: *w*_*pp*_ (weight of positive privileged samples), *w*_*pup*_ (weight of positive unprivileged samples), *w*_*np*_ (weight of negative privileged samples), and *w*_*nup*_ (weight of negative unprivileged samples), as outlined below.


(1) $$\begin{array}{l} w_{pp}=\frac{N_{p}}{N_{total}}\times \frac{N_{pos}}{N_{pp}} \end{array}$$

(2) $$\begin{array}{l} w_{pup}=\frac{N_{up}}{N_{total}}\times \frac{N_{pos}}{N_{pup}} \end{array}$$

(3) $$\begin{array}{l} w_{np}=\frac{N_{p}}{N_{total}}\times \frac{N_{neg}}{N_{np}} \end{array}$$

(4) $$\begin{array}{l} w_{nup}=\frac{N_{up}}{N_{total}}\times \frac{N_{neg}}{N_{up}} \end{array}$$

where

*N*_*p*_: the number of samples in the privileged group.

*N*_*pp*_: the number of samples with the positive class in the privileged group.

*N*_*np*_: the number of samples with the negative class in the privileged group.

*N*_*up*_: the number of samples in the unprivileged group.

*N*_*pup*_: the number of samples with the positive class in the unprivileged group.

*N*_*nup*_: the number of samples with the negative class in the unprivileged group.

*N*_*pos*_: the number of samples with the positive class.

*N*_*neg*_: the number of samples with the negative class.

*N*_*total*_: the number of samples.

## 3 Experiment

### 3.1 Datasets

This study utilized the Adult Income and the COMPAS datasets and applied TabDDPM to generate new synthetic data, aiming to assess the combined effectiveness of data augmentation and sample reweighting in mitigating fairness issues.

**Adult income dataset:** The dataset consists of 48, 842 samples with 14 attributes, designed to predict whether an individual's income exceeds $50K/year based on census data (Becker and Kohavi, 1996). It was divided into training (28, 048 samples), testing (16, 281 samples), and validation (6, 513 samples) sets. The attributes were categorized into 8 categorical and 6 numerical features.

**COMPAS dataset:** The dataset consists of 7, 214 samples with 53 attributes, 18 attributes were used for generating data, which was used to determine whether a person would recidivate after two years. It was divided into training (4, 311 samples), testing (1, 724 samples), and validation (1, 150 samples) sets. The attributes were categorized into 211 categorical and 13 numerical features.

**Figure 3:**
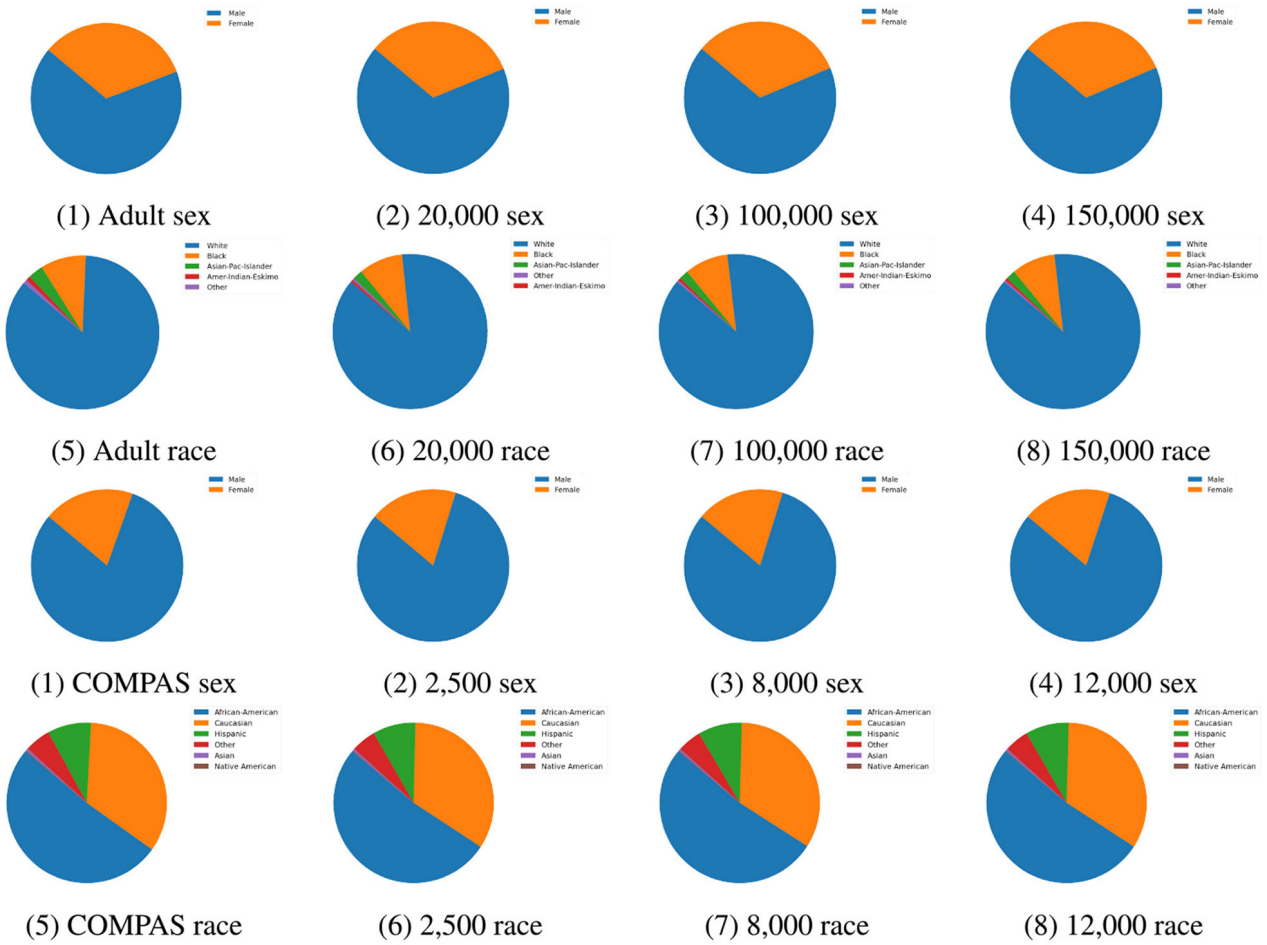
Attribute distribution comparison between original data and synthetic data for Adult Income and COMPAS datasets. The attributes sex and race for both datasets were compared.

**Synthetic dataset:** TabDDPM was employed to generate synthetic samples to implement data augmentation, enhancing both AI fairness and classification performance. Synthetic data was added the Adult Income training set in sample sizes of 20, 000, 100, 000, and 150, 000. Similarly, synthetic data was added the COMPAS training set in sample sizes 2, 500, 8, 000, and 12, 000. As show in Figure 3, the distributions of synthetic data closely resemble the original data across sample sizes. Furthermore, the synthetic data is free of missing values, improving overall data quality. These observations suggest that synthetic data is a promising way for data augmentation, particularly in terms of maintaining data quality.

### 3.2 Evaluation metrics

This paper utilized five evaluation metrics to determine the effectiveness of reweighting samples for mitigating bias.

Disparate Impact (DI) refers to the unintentional bias that can occur when predictions result in varying error rates or outcomes across different demographic groups, where certain attributes like race, sex, religion, and age are considered protected. This bias may arise from training models on biased data or from the model itself being discriminatory. In this study, Disparate Impact is defined as the differential effects on prediction outcomes.


(5) $$\begin{array}{l} DI=\frac{p_{pup}}{p_{pp}} \end{array}$$

where *p*_*pup*_ presents the prediction probability for unprivileged samples with positive predictions, while *p*_*pp*_ denotes the prediction probability for privileged samples with positive predictions. A disparate impact value approaching 0 indicates bias in favor of the privileged group, while a value >1 indicates bias in favor of the unprivileged group. A value of 1 reflects perfect fairness in the predictions (Feldman et al., 2015).

**Average odds difference (AOD)** measures the average difference in false positive rates (FPR) and true positive rates (TPR) between unprivileged and privileged groups. It is calculated as:


(6) $$\begin{array}{l} AOD=\frac{\left(FPR_{up}-FPR_{p}\right)+\left(TPR_{up}-TPR_{p}\right)}{2} \end{array}$$

where *FPR*_*up*_ and *FPR*_*p*_ represent the False Positive Rates for unprivileged and privileged samples, respectively, while *TPR*_*up*_ and *TPR*_*p*_ represent the True Positive Rates for unprivileged and privileged samples. An AOD value of 0 indicates perfect fairness. A positive AOD suggests bias in favor of the unprivileged group, while a negative AOD indicates bias in favor of the privileged group.

**Statistical parity difference (SPD)** is to calculate the difference between the ratio of favorable outcomes in unprivileged and privileged groups. It is defined by


(7) $$\begin{array}{l} SPD=p_{pup}-p_{pp} \end{array}$$

A score below 0 suggests benefits for the unprivileged group, while a score above 0 implies benefits for the privileged group. A score of 0 indicates that both groups receive equal benefits.

**Equal opportunity difference (EOD)** assesses whether all groups have an equal chance of benefiting from predictions. EOD focuses on the True Positive Rate (TPR), which reflects the model's ability to correctly identify positives in both unprivileged and privileged groups. It is defined as follows:


(8) $$\begin{array}{l} EOD=TPR_{up}-TPR_{p} \end{array}$$

A value of 0 signifies perfect fairness. A positive value indicates bias in favor of the unprivileged group, while a negative value indicates bias in favor of the privileged group.

**Theil index (TI)** is also called the entropy index which measures both the group and individual fairness. It is defined by


(9) $$\begin{array}{l} TI=\frac{1}{n}\overset{n}{\underset{i=1}{\sum }}\frac{b_{i}}{\mu }ln\frac{b_{i}}{\mu } \end{array}$$

where $b_{i}=\hat{y_{i}}-y_{i}+1$ and μ is the average of *b*_*i*_. A lower absolute value of TI value in this context would indicate a more equitable distribution of classification outcomes, while a higher absolute value suggests greater disparity.

Table 1 presents the fairness ranges and levels of various evaluation metrics. If the values of evaluation metrics fall into these ranges, it indicates that the machine learning models perform classification without bias. For the DI, the commonly accepted fairness range is [0.8, 1.25]. If the selection rate for the protected group is at least 80% of the selection rate for the unprotected group, the disparity is generally considered fair. Conversely, a DI >1.25 indicates a reverse disparity, potentially disadvantaging the unprotected group. For AOD, the range is the same as that of SPD and EOD. AOD = 0 signifies no difference in TPR (True Positive Rate) or FPR (False Positive Rate) between groups, indicating perfect fairness. The acceptable fairness range for AOD is typically [−0.1, 0.1], where AOD >0.1 indicates bias in favor of the protected group. AOD < −0.1 indicates bias in favor of the unprotected group. For the TI, higher values indicate greater unfairness, while TI = 0 represents perfect fairness.

**Table 1:** Fairness ranges of various evaluation metrics.

**Metrics**	**Fairness ranges**
DI	0.80 ≤ *fair* ≤ 1.25
AOD	−0.10 ≤ *fair* ≤ 0.10
SPD	−0.10 ≤ *fair* ≤ 0.10
EOD	−0.10 ≤ *fair* ≤ 0.10
TI	0 ≤ *fair* ≤ 0.25

### 3.3 Results and discussion

To comprehensively validate the proposed method, we conduct extensive experiments in that regard of two protect attributes, namely Race and Sex, to examine the effectiveness of bias mitigation.

#### 3.3.1 Case study on adult income datasets

**Race:** Table 2 presents a performance comparison across all outputs before reweighting samples, using one classification metric, BA, and five fairness metrics–SPD, AOD, DI, EOD, and TI–on the Adult Income dataset, focusing on the protected attribute of *Race*. Generally, before reweighting samples to mitigate bias, augmenting the training data does not appear to effectively reduce bias. For example, the SPD value for GNB increases with the addition of synthetic samples, indicating that synthetic data may actually exacerbate bias. Additionally, the performance of LR in terms of classification and bias mitigation remains relatively unchanged, as reflected in the BA, AOD, and TI values. However, it is observed that the fairness of KNN is improved regarding the changes of values of PSD, AOD, DI, EOD, and TI.

**Table 2:** Performance comparison between all outputs before and after reweighting through one classification metric BA and fairness metrics including SPD, AOD, DI, EOD, and TI on Adult income dataset regarding the protected attribute *Race*.

**Performance before reweighting samples (original)**							**Performance after reweighting samples (original)**						
**Model**	**BA**	**SPD**	**AOD**	**DI**	**EOD**	**TI**	**Model**	**BA**	**SPD**	**AOD**	**DI**	**EOD**	**TI**
DT	0.7426	−0.2416	−0.1959	0.4196	−0.2026	0.1130	DT	1.0	−0.1066	0.0	0.5863	0.0	0.0
GNB	0.7416	−0.2952	−0.2623	0.3252	−0.2872	0.1111	GNB	0.7432	−0.1147	−0.0252	0.7379	0.0310	0.1058
KNN	0.7390	−0.1904	−0.1409	0.4882	−0.1416	0.1207	KNN	0.7390	−0.1904	−0.1409	0.4882	−0.1416	0.1207
LR	0.7437	−0.2435	−0.1966	0.4122	−0.2020	0.1129	LR	0.7311	−0.0523	0.0419	0.8508	0.1083	0.1247
RF	0.7471	−0.2014	−0.1336	0.5380	−0.1097	0.1066	RF	0.7447	−0.1072	−0.0201	0.7449	0.0321	0.1081
**Performance before reweighting samples (20,000)**							**Performance after reweighting samples (20,000)**						
**Model**	**BA**	**SPD**	**AOD**	**DI**	**EOD**	**TI**	**Model**	**BA**	**SPD**	**AOD**	**DI**	**EOD**	**TI**
DT	0.7441	−0.2182	−0.1763	0.4791	−0.1791	0.1117	DT	1.0	−0.0913	0.0	0.6444	0.0	0.0
GNB	0.7373	−0.3107	−0.2975	0.2965	−0.3417	0.1129	GNB	0.7354	−0.0984	−0.0358	0.7574	−0.0051	0.1151
KNN	0.7303	**−0.1021**	**−0.0262**	**0.7544**	**0.0262**	**0.1156**	KNN	0.7293	−0.1067	−0.0302	0.7461	0.0236	0.1153
LR	0.7432	−0.2157	−0.1705	0.5099	−0.1678	0.1082	LR	0.7415	−0.0446	0.0204	0.8978	0.0526	0.1052
RF	0.7440	−0.1414	−0.0802	0.6776	−0.0539	0.1063	RF	0.7440	−0.0946	−0.0243	0.7842	0.0157	0.1051
**Performance before reweighting samples (100,000)**							**Performance after reweighting samples (100,000)**						
**Model**	**BA**	**SPD**	**AOD**	**DI**	**EOD**	**TI**	**Model**	**BA**	**SPD**	**AOD**	**DI**	**EOD**	**TI**
DT	0.7496	−0.1260	−0.0573	0.7148	−0.0313	0.1020	DT	1.0	−0.1031	0.0	0.5958	0.0	0.0
GNB	0.7345	−0.3976	−0.3794	0.2321	−0.4205	0.1003	GNB	0.7440	−0.1133	−0.0455	0.7462	−0.0191	0.1032
KNN	0.7233	−0.1619	−0.1009	0.5979	−0.0778	0.1214	KNN	0.7234	−0.1602	−0.0980	0.6022	−0.0732	0.1213
LR	0.7504	−0.2324	−0.1788	0.4756	−0.1741	0.1039	LR	0.7487	−0.1044	−0.0388	0.7554	−0.0170	0.1045
RF	0.7503	−0.1699	−0.1084	0.6144	−0.0926	0.1029	RF	0.7489	−0.1092	−0.0427	0.7525	−0.0196	0.1020
**Performance before reweighting samples (150,000)**							**Performance before reweighting samples (150,000)**						
**Model**	**BA**	**SPD**	**AOD**	**DI**	**EOD**	**TI**	**Model**	**BA**	**SPD**	**AOD**	**DI**	**EOD**	**TI**
DT	0.7473	−0.2262	−0.1895	0.4790	−0.2081	0.1066	DT	1.0	−0.1020	0.0	0.5995	0.0	0.0
GNB	0.7262	−0.4150	−0.3996	0.2226	−0.4418	0.1006	GNB	0.7296	**−0.0906**	**−0.0344**	**0.8225**	**−0.0202**	**0.0955**
KNN	0.7382	−0.1237	−0.0797	0.6983	−0.0858	0.1126	KNN	0.7382	−0.1237	−0.0797	0.6983	−0.0858	0.1126
LR	0.7468	−0.2180	−0.1782	0.5007	−0.1922	0.1062	LR	0.7414	**−0.0127**	**0.0340**	**0.9709**	**0.0299**	**0.1037**
RF	0.7476	−0.1862	−0.1379	0.5713	−0.1399	0.1056	RF	0.7461	**−0.0895**	**−0.0300**	**0.7939**	**−0.0158**	**0.1040**

It presents the performances of adding different numbers of synthetic samples to the original data, including 20, 000, 100, 000, and 150, 000. The bold values highlight the outstanding performance regarding AI fairness of ML models.

On the other hand, after reweighting the samples, bias is mitigated for most ML models, as indicated by improvements in SPD, AOD, and TI values. For instance, the bias in LR significantly decreases across all fairness metrics–SPD, AOD, DI, EOD, and TI. Similar trends are observed for other models like DT, GNB, and RF. Moreover, when more synthetic samples are added to the original training data, bias is further reduced, particularly in the case of the (150, 000) sample size, as shown in the improved performance of LR and RF.

**Sex:** Table 3 presents a performance comparison of all outputs before reweighting samples, using one classification metric (BA) and five fairness metrics (SPD, AOD, DI, EOD, and TI) on the Adult Income dataset, focusing on the protected attribute *Sex*. Before reweighting samples to address bias, similar patterns are observed: augmenting the training data does not effectively reduce bias, as shown consistently across ML models like KNN and LR, particularly in fairness metrics such as SPD and AOD for the cases of the (150, 000) sample size.

**Table 3:** Performance comparison between all outputs before and after reweighting through one classification metric BA and fairness metrics including SPD, AOD, DI, EOD, and TI on Adult income dataset regarding the protected attribute *Sex*.

**Performance before reweighting samples (original)**							**Performance after reweighting samples (original)**						
**Model**	**BA**	**SPD**	**AOD**	**DI**	**EOD**	**TI**	**Model**	**BA**	**SPD**	**AOD**	**DI**	**EOD**	**TI**
DT	0.7426	−0.3608	−0.3204	0.2785	−0.3775	0.1130	DT	1.0	−0.1910	0.0	0.3740	0.0	0.0
GNB	0.7416	−0.3353	−0.2805	0.3369	−0.3184	0.1111	GNB	0.7209	−0.0861	0.0073	0.7997	0.0203	0.1192
KNN	0.7390	−0.3983	−0.4075	0.1616	−0.5311	0.1207	KNN	0.7390	−0.3983	−0.4075	0.1616	−0.5311	0.1207
LR	0.7437	−0.3580	−0.3181	0.2794	−0.3769	0.1129	LR	0.7134	−0.0705	0.0188	0.7785	0.0293	0.1401
RF	0.7471	−0.3777	−0.3292	0.2884	−0.3763	0.1066	RF	0.7271	−0.1386	−0.0638	0.7220	−0.0774	0.1065
**Performance before reweighting samples (20,000)**							**Performance after reweighting samples (20,000)**						
**Model**	**BA**	**SPD**	**AOD**	**DI**	**EOD**	**TI**	**Model**	**BA**	**SPD**	**AOD**	**DI**	**EOD**	**TI**
DT	0.7441	**−0.2182**	**−0.1763**	**0.4791**	**−0.1791**	**0.1117**	DT	1.0	−0.1957	0.0	0.3657	0.0	0.0
GNB	0.7373	−0.3600	−0.3072	0.3044	−0.3461	0.1129	GNB	0.7143	−0.0984	−0.0215	0.7791	−0.0303	0.1205
KNN	0.7303	−0.2969	−0.2315	0.4052	−0.2528	0.1156	KNN	0.7293	−0.3028	−0.2366	0.4005	−0.2556	0.1153
LR	0.7432	−0.3905	−0.3344	0.2753	−0.3699	0.1082	LR	0.7173	−0.0100	−0.0244	0.8020	−0.0369	0.1071
RF	0.7440	−0.3993	−0.3379	0.2740	−0.3656	0.1063	RF	0.7188	−0.1065	−0.0318	0.7920	−0.0458	0.1053
**Performance before reweighting samples (100,000)**							**Performance after reweighting samples (100,000)**						
**Model**	**BA**	**SPD**	**AOD**	**DI**	**EOD**	**TI**	**Model**	**BA**	**SPD**	**AOD**	**DI**	**EOD**	**TI**
DT	0.7496	−0.4149	−0.3538	0.2580	−0.3871	0.1020	DT	1.0	−0.2029	0.0	0.3394	0.0	0.0
GNB	0.7345	−0.4182	−0.3477	0.3047	−0.3588	0.1003	GNB	0.7195	−0.1084	−0.0220	0.7711	−0.0242	0.1123
KNN	0.7233	−0.3791	−0.3225	0.2478	−0.3491	0.1214	KNN	0.7234	−0.3795	−0.3229	0.2477	−0.3495	0.1213
LR	0.7504	−0.4016	−0.3460	0.2601	−0.3883	0.1039	LR	0.7249	−0.1089	−0.0196	0.7639	−0.0216	0.1124
RF	0.7503	−0.4047	−0.3447	0.2631	−0.3806	0.1029	RF	0.7192	**−0.0633**	**0.0257**	**0.8602**	**−0.0240**	**0.1137**
**Performance before reweighting samples (150,000)**							**Performance before reweighting samples (150,000)**						
**Model**	**BA**	**SPD**	**AOD**	**DI**	**EOD**	**TI**	**Model**	**BA**	**SPD**	**AOD**	**DI**	**EOD**	**TI**
DT	0.7473	−0.4031	−0.3579	0.2494	−0.4098	0.1066	DT	1.0	−0.1956	0.0	0.3600	0.0	0.0
GNB	0.7262	−0.3525	−0.2867	0.4090	−0.3007	0.1006	GNB	0.7189	−0.0964	−0.0245	0.7967	−0.0433	0.1118
KNN	0.7382	−0.3494	−0.2938	0.3124	−0.3296	0.1126	KNN	0.7382	−0.3493	−0.2938	0.3124	−0.3296	0.1126
LR	0.7468	−0.3961	−0.3487	0.2641	−0.3977	0.1062	LR	0.7200	−0.0976	−0.0254	0.8069	−0.0441	0.1048
RF	0.7476	−0.3916	−0.3383	0.2725	−0.3799	0.1056	RF	0.7230	−0.1065	−0.0289	0.7914	−0.0411	0.1032

The bold values highlight the outstanding performance regarding AI fairness of ML models.

However, after reweighting the samples, bias is reduced in most ML models, as indicated by improvements in SPD, AOD, and TI. For example, significant bias reductions are observed across all fairness metrics–SPD, AOD, DI, EOD, and TI–for models like LR, GNB, DT, and RF. Moreover, when additional synthetic samples are added to the original training data, bias is further mitigated, particularly in the case of GNB at the (150, 000) sample size.

**Figure 4:**
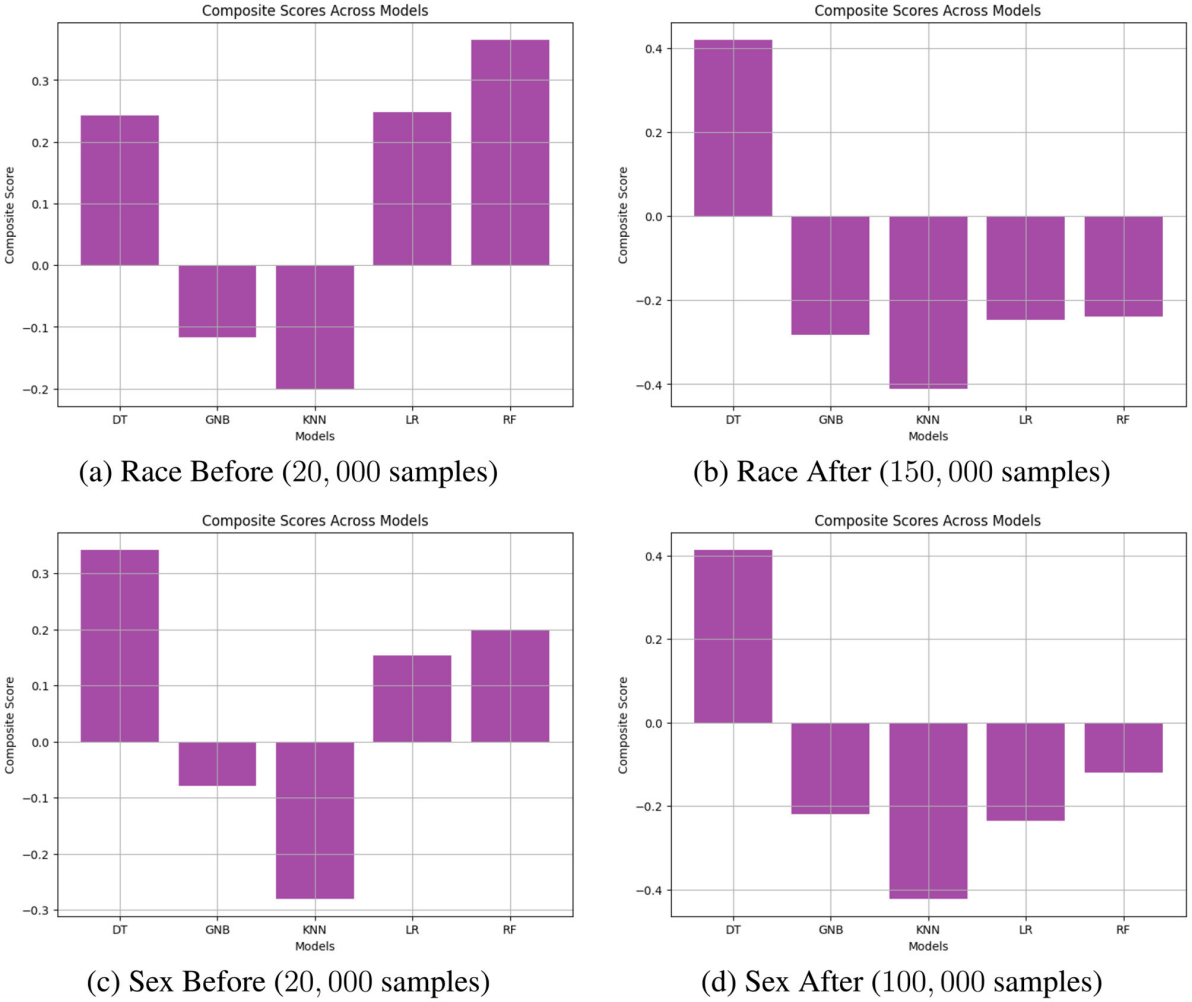
Navigating trade-off between BA and fairness metrics on Adult Income datasets including DI, AOD, SPD, EOD, and TI across all models through calculating composite scores. **(A)** Race Before (20, 000 samples). **(B)** Race After (150, 000 samples). **(C)** Sex Before (20, 000 samples). **(D)** Sex After (100, 000 samples).

Furthermore, to address the trade-off between BA and fairness metrics such as DI, AOD, SPD, EOD, and TI, we introduce the composite score defined below,


(10) $$\begin{array}{c} Score_{CS}=w_{BA}\times BA-w_{SPD}\times |SPD|-w_{AOD}\times |AOD|\\ \;-w_{EOD}\times |EOD|-w_{TI}\times TI-w_{DI}\times DI \end{array}$$

where the weights *w*_*BA*_, *w*_*SPD*_, *w*_*AOD*_, *w*_*EOD*_, *w*_*TI*_, and *w*_*DI*_ are adjusted depending on the scenario. In this study, we set *w*_*BA*_ = 0.5, while the other weights are equally set to 0.1. Additionally, all metric values are normalized, and a higher *Score*_*CS*_ indicates better model performance. Figure 4 illustrates an example of navigating the trade-off by calculating the composite score across all models. Figures 4A, B are based on the results of performance before reweighting samples (20,000) and after reweighting samples (100,000) for the protected attribute Race. Similarly, Figures 4C, D are based on the results of performance before reweighting samples (20,000) and after reweighting samples (150,000) for the protected attribute *Race*. The results reveal that for the protected attribute *Race*, RF and LR generally outperformed other models in terms of *Score*_*CS*_, as shown in Figures 4A, B, respectively. In the case of the protected attribute *Sex*, DT demonstrated better performance compared to the other models.

**Figure 5:**
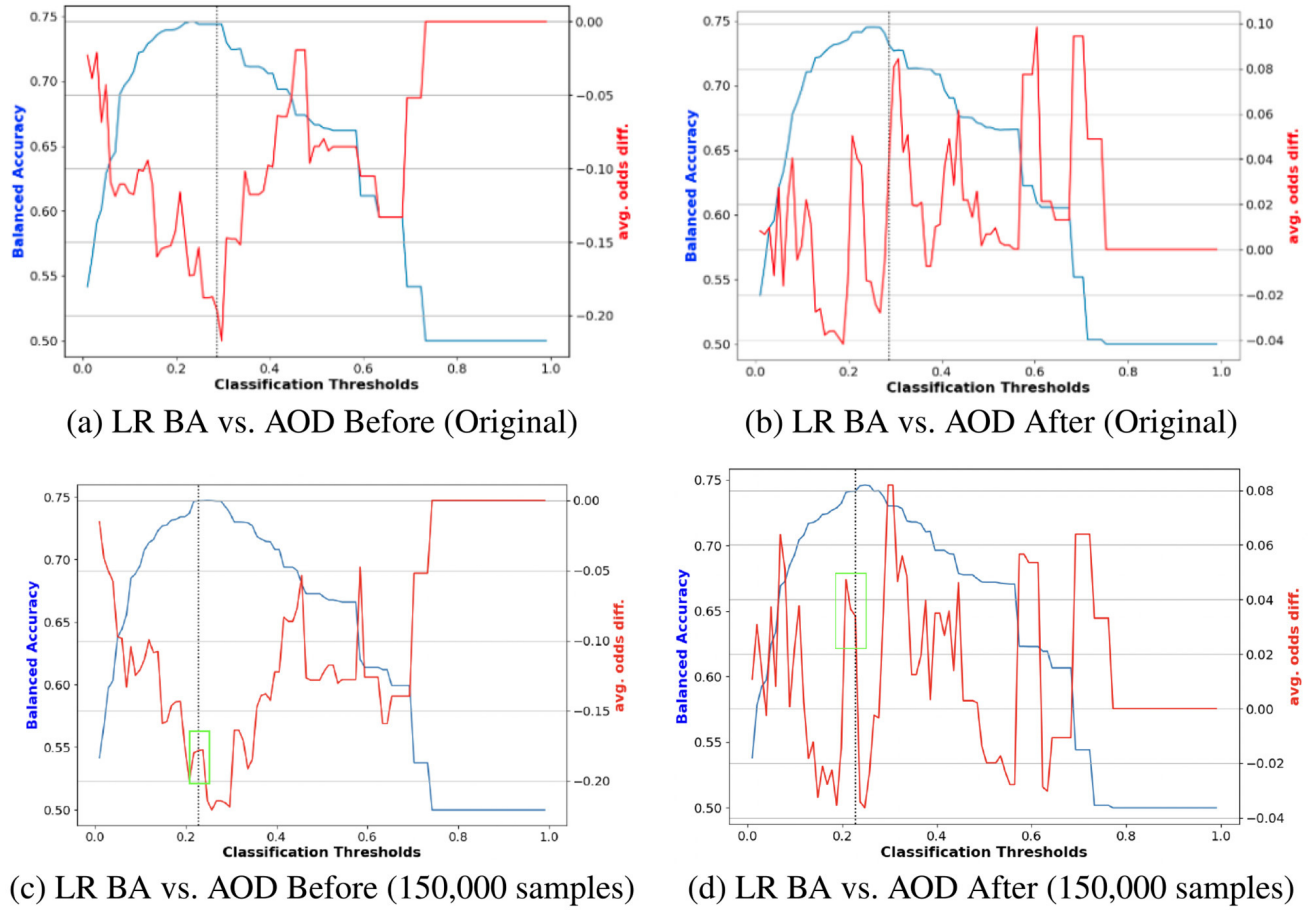
Performance comparison of BA and AOD in augmenting training data with 150, 000 synthetic samples on the Adult Income dataset, considering the protected attribute *Race* for LR. Subfigures **(A)** and **(C)** illustrate the performance comparison between the original training data and the augmented training data (with 150, 000 synthetic samples) before reweighting the samples. In contrast, Subfigures **(B)** and **(D)** present the comparison after reweighting the samples. **(A)** LR BA vs. AOD Before (Original). **(B)** LR BA vs. AOD After (Original). **(C)** LR BA vs. AOD Before (150,000 samples). **(D)** LR BA vs. AOD After (150,000 samples).

**Figure 6:**
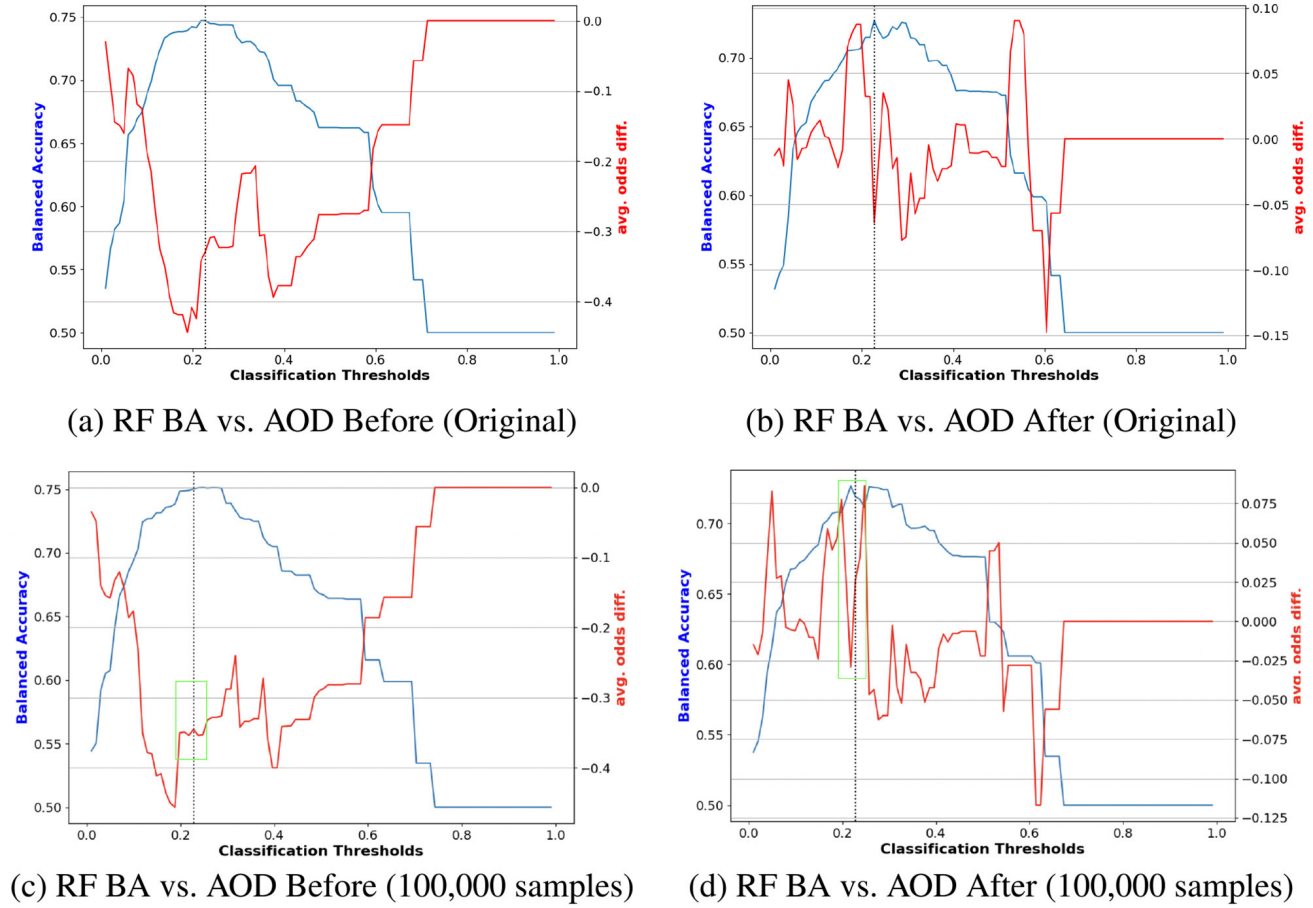
Performance comparison of BA and AOD in augmenting training data with 100, 000 synthetic samples on the Adult Income dataset, considering the protected attribute *Sex* for RF. Subfigures **(A)** and **(C)** illustrate the performance comparison between the original training data and the augmented training data (with 100, 000 synthetic samples) before reweighting the samples. In contrast, Subfigures **(B)** and **(D)** present the comparison after reweighting the samples. **(A)** RF BA vs. AOD Before (Original). **(B)** RF BA vs. AOD After (Original). **(C)** RF BA vs. AOD Before (100,000 samples). **(D)** RF BA vs. AOD After (100,000 samples).

Finally, Figures 5, 6 illustrate the impact of synthetic data on model fairness and accuracy by comparing the performance of models trained on the original data to those trained on augmented datasets incorporating synthetic samples. In Figures 5A, C show performance comparisons between the original training data and the augmented data (with 150, 000 synthetic samples) before reweighting. The results reveal that adding 150, 000 synthetic samples improves the stability of LR fairness, particularly evident in the stable AOD values near the optimal trade-off point, marked by the green box in Figure 5B. Notably, this augmentation does not significantly affect BA values.

Conversely, Figures 5B, D demonstrate the effects of reweighting on the augmented training data. Reweighting further emphasizes the benefits of synthetic augmentation by narrowing the range of absolute AOD values from [–0.25, 0] to [–0.04, 0.08]. Near the optimal trade-off point highlighted in the green box of Figures 5D, the augmented data exhibits greater stability, as evidenced by the less pronounced changes in AOD values.

A similar trend is observed in Figure 6. Figures 6A, C compare the original data and the augmented data (with 100, 000 synthetic samples) before reweighting. The stable AOD values in the green box of Figure 6C suggest that the augmented data enhances fairness stability for RF. Figures 6B, D reveal that reweighting further improves fairness, as indicated by the reduced range of absolute AOD values, reinforcing the notion that combining reweighting with data augmentation effectively enhances model fairness.

#### 3.3.2 Case study on COMPAS datasets

**Race:** Table 4 presents a performance comparison on the COMPAS dataset, focusing on the protected attribute *Race*. Prior to reweighting, adding 12, 000 synthetic samples to the training set significantly enhanced the fairness of RF in terms of fairness metrics such as SPD and DI, albeit at the cost of BA performance. After reweighting, the inclusion of synthetic samples further improved the fairness of multiple models in the training dataset.

**Table 4:** Performance comparison between all outputs before and after reweighting through one classification metric BA and fairness metrics including SPD, AOD, DI, EOD, and TI on COMPAS dataset regarding the protected attribute *Race*.

**Performance before reweighting samples (original)**							**Performance after reweighting samples (original)**						
**Model**	**BA**	**SPD**	**AOD**	**DI**	**EOD**	**TI**	**Model**	**BA**	**SPD**	**AOD**	**DI**	**EOD**	**TI**
DT	0.6586	−0.1516	−0.0970	0.7791	−0.1212	0.1835	DT	1.0	−0.1769	0.0	0.7060	0.0	0.0
GNB	0.6553	−0.2493	−0.1994	0.6155	−0.1980	0.2382	GNB	0.6437	−0.1782	−0.1318	0.6926	−0.1251	0.2594
KNN	0.6414	−0.2139	−0.1727	0.7282	−0.1131	0.1607	KNN	0.6311	−0.3432	−0.3105	0.5945	−0.2391	0.1762
LR	0.6774	−0.2494	−0.1927	0.6600	−0.1877	0.1774	LR	0.6342	−0.0546	0.1042	1.1062	0.1215	0.2257
RF	0.6432	−0.1539	−0.1057	0.6873	−0.1166	0.3003	RF	0.6234	0.1459	0.1953	1.4012	0.1977	0.2867
**Performance before reweighting samples (2,500)**							**Performance after reweighting samples (2,500)**						
**Model**	**BA**	**SPD**	**AOD**	**DI**	**EOD**	**TI**	**Model**	**BA**	**SPD**	**AOD**	**DI**	**EOD**	**TI**
DT	0.6636	−0.2480	−0.2182	0.6604	−0.1588	0.2106	DT	1.0	−0.1283	0.0	0.7940	0.0	0.0
GNB	0.6605	−0.2715	−0.2388	0.5787	−0.2068	0.2734	GNB	0.6495	−0.1913	−0.1603	0.6768	−0.1275	0.2838
KNN	0.6554	−0.3124	−0.2783	0.4966	−0.2686	0.3074	KNN	0.6560	−0.4031	−0.3723	0.4333	−0.3791	0.2848
LR	0.6638	−0.2705	−0.2408	0.6196	−0.1832	0.2300	LR	0.6483	−0.0122	0.0424	1.0248	0.1006	0.2701
RF	0.6611	−0.2148	−0.1842	0.7049	−0.1307	0.2017	RF	0.6414	−0.0625	−0.0925	1.1290	0.1456	0.2604
**Performance before reweighting samples (8,000)**							**Performance after reweighting samples (8,000)**						
**Model**	**BA**	**SPD**	**AOD**	**DI**	**EOD**	**TI**	**Model**	**BA**	**SPD**	**AOD**	**DI**	**EOD**	**TI**
DT	0.6468	−0.0895	−0.0461	0.8310	−0.0568	0.2749	DT	1.0	−0.1469	0.0	0.7615	0.0	0.0
GNB	0.6399	−0.2965	−0.2562	0.4838	−0.2953	0.3283	GNB	0.6332	−0.1917	−0.1518	0.6266	−0.1858	0.3325
KNN	0.6353	−0.2984	−0.2637	0.4961	−0.2674	0.3016	KNN	0.6293	−0.1861	−0.1509	0.6602	−0.1539	0.3123
LR	0.6547	−0.1959	−0.1553	0.7193	−0.1416	0.2082	LR	0.6453	−0.0331	−0.0203	0.9403	−0.0061	0.2409
RF	0.6523	−0.1770	−0.1356	0.7496	−0.1304	0.1981	RF	0.6385	**0.0167**	**0.0600**	**1.0297**	**0.0477**	**0.2244**
**Performance before reweighting samples (12,000)**							**Performance before reweighting samples (12,000)**						
**Model**	**BA**	**SPD**	**AOD**	**DI**	**EOD**	**TI**	**Model**	**BA**	**SPD**	**AOD**	**DI**	**EOD**	**TI**
DT	0.5869	−0.2013	−0.1846	0.5897	−0.2166	0.4046	DT	1.0	−0.0794	0.0	0.8665	0.0	0.0
GNB	0.5895	−0.1650	−0.1524	0.6982	−0.1475	0.3484	GNB	0.5871	−0.1445	−0.1317	0.7346	−0.1302	0.3434
KNN	0.5780	−0.1238	−0.1115	0.8329	−0.1160	0.2128	KNN	0.5662	**0.0438**	**0.0543**	**1.0617**	**−0.0568**	**0.1776**
LR	0.5952	−0.3114	−0.2988	0.5477	−0.2938	0.3075	LR	0.5996	0.0320	0.0428	1.0607	0.0847	0.2760
RF	0.6038	−0.0308	−0.0170	0.9385	0.0034	0.3144	RF	0.6121	0.1153	0.1315	1.2704	0.1508	0.3003

It presents the performances of adding different numbers of synthetic samples to the original data, including 2, 500, 8, 000, and 12, 000. The bold values highlight the outstanding performance regarding AI fairness of ML models.

**Sex:** Table 5 presents a performance comparison on the COMPAS dataset, focusing on the protected attribute *Sex*. Adding synthetic samples to the training set alone does not appear to improve model fairness. However, combining reweighting with synthetic sample augmentation proves effective in enhancing fairness.

**Table 5:** Performance comparison between all outputs before and after reweighting through one classification metric BA and fairness metrics including SPD, AOD, DI, EOD and TI on COMPAS dataset regarding the protected attribute *Sex*.

**Performance before reweighting samples (original)**							**Performance after reweighting samples (original)**						
**Model**	**BA**	**SPD**	**AOD**	**DI**	**EOD**	**TI**	**Model**	**BA**	**SPD**	**AOD**	**DI**	**EOD**	**TI**
DT	0.6586	−0.1637	−0.1340	0.7759	−0.0597	0.1835	DT	1.0	−0.1383	0.0	0.7732	0.0	0.0
GNB	0.6553	−0.4129	−0.3877	0.5066	−0.3090	0.2382	GNB	0.6581	−0.1998	−0.1720	0.7154	−0.0899	0.2146
KNN	0.6414	−0.2336	−0.2095	0.7256	−0.1350	0.1607	KNN	0.6311	−0.2551	−0.2318	0.7003	−0.1708	0.1762
LR	0.6774	−0.2724	−0.2439	0.6631	−0.1392	0.1774	LR	0.6562	−0.1188	−0.0946	0.8342	0.0111	0.1730
RF	0.6432	−0.3759	−0.3484	0.4700	−0.3002	0.3003	RF	0.6585	−0.1615	−0.1279	0.7081	−0.0760	0.2776
**Performance before reweighting samples (2,500)**							**Performance after reweighting samples (2,500)**						
**Model**	**BA**	**SPD**	**AOD**	**DI**	**EOD**	**TI**	**Model**	**BA**	**SPD**	**AOD**	**DI**	**EOD**	**TI**
DT	0.6636	−0.1766	−0.1594	0.7557	−0.1257	0.2106	DT	1.0	−0.0728	0.0	0.8795	0.0	0.0
GNB	0.6605	−0.3307	−0.3153	0.5577	−0.2769	0.2734	GNB	0.6463	−0.0525	−0.0401	0.8930	0.0088	0.3038
KNN	0.6554	−0.2076	−0.1917	0.6535	−0.1576	0.3074	KNN	0.6560	−0.2076	−0.1917	0.6535	−0.1576	0.3074
LR	0.6638	−0.1926	−0.1744	0.7261	−0.1463	0.2300	LR	0.6538	**−0.0325**	**−0.0210**	**0.9448**	**0.0387**	**0.2272**
RF	0.6611	−0.1852	−0.1701	0.7523	−0.1269	0.2017	RF	0.6523	0.0188	0.0350	1.0333	0.0687	0.2181
**Performance before reweighting samples (8,000)**							**Performance after reweighting samples (8,000)**						
**Model**	**BA**	**SPD**	**AOD**	**DI**	**EOD**	**TI**	**Model**	**BA**	**SPD**	**AOD**	**DI**	**EOD**	**TI**
DT	0.6468	−0.2197	−0.1762	0.6659	−0.1607	0.2749	DT	1.0	−0.1734	0.0	0.7417	0.0	0.0
GNB	0.6399	−0.3116	−0.2718	0.5236	−0.2579	0.3283	GNB	0.6192	−0.0371	−0.0034	0.9050	0.0234	0.3682
KNN	0.6353	−0.3909	−0.3487	0.4691	−0.3533	0.3216	KNN	0.6293	−0.2432	−0.1971	0.6182	−0.2136	0.3123
LR	0.6547	−0.1886	−0.1516	0.7438	−0.1022	0.2082	LR	0.6432	0.0252	0.0656	1.0457	0.1025	0.2206
RF	0.6523	−0.2428	−0.2078	0.6970	−0.1576	0.1981	RF	0.6394	**0.0015**	**0.0451**	**1.0025**	**0.0645**	**0.2026**
**Performance before reweighting samples (12,000)**							**Performance before reweighting samples (12,000)**						
**Model**	**BA**	**SPD**	**AOD**	**DI**	**EOD**	**TI**	**Model**	**BA**	**SPD**	**AOD**	**DI**	**EOD**	**TI**
DT	0.5870	−0.3378	−0.3213	0.4793	−0.3453	0.4046	DT	1.0	−0.0742	0.0	0.8781	0.0	0.0
GNB	0.5895	−0.3767	−0.3694	0.5035	−0.3442	0.3484	GNB	0.5830	−0.1196	−0.1104	0.7677	−0.0956	0.3756
KNN	0.5780	−0.2281	−0.2252	0.7333	−0.1834	0.2128	KNN	0.5662	−0.1450	−0.1426	0.8304	−0.1061	0.1776
LR	0.5952	−0.2848	−0.2771	0.6137	−0.2484	0.3075	LR	0.5903	−0.0703	−0.0630	0.8655	−0.0325	0.3365
RF	0.6038

The bold values highlight the outstanding performance regarding AI fairness of ML models.

**Figure 7:**
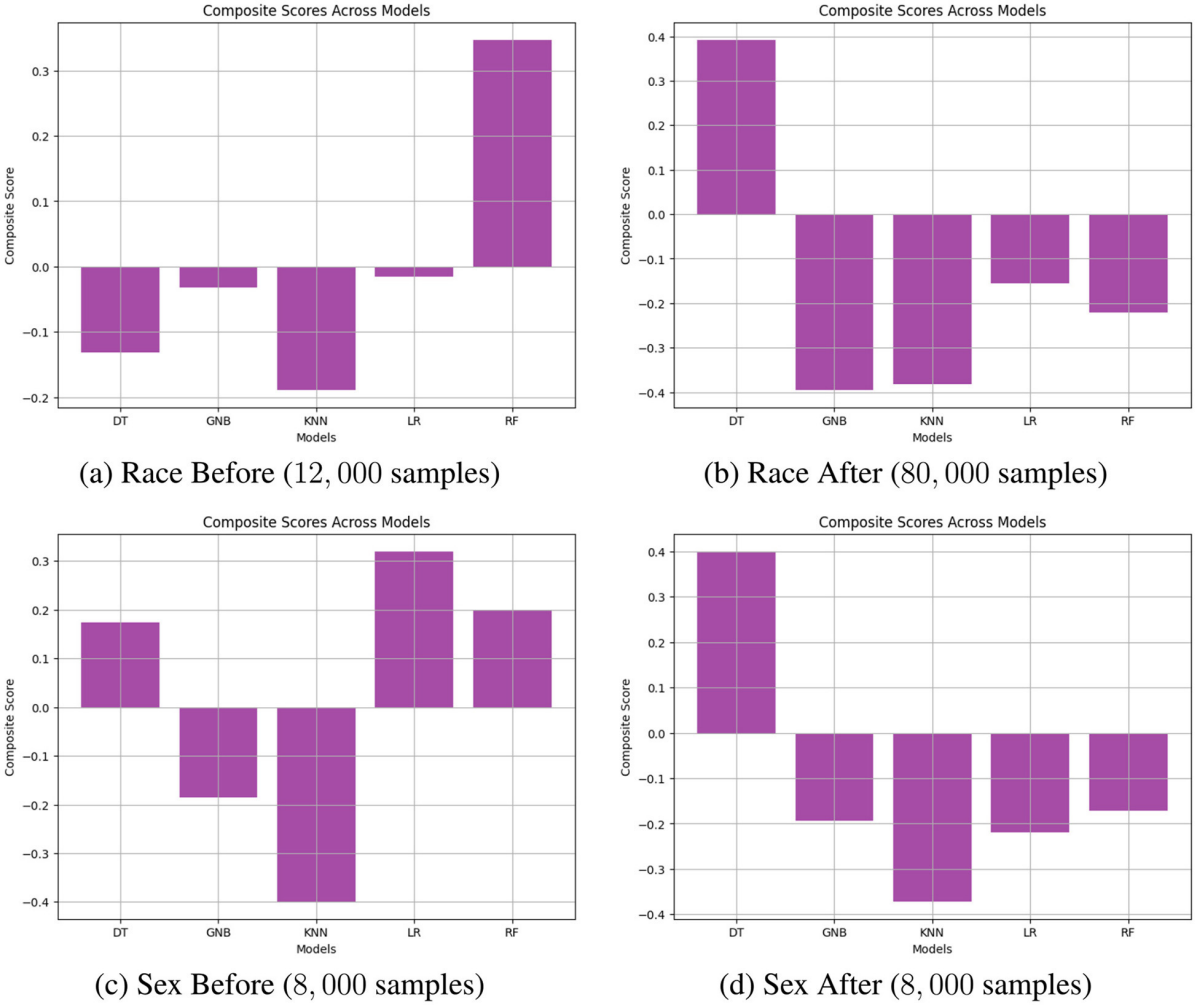
Navigating trade-off between BA and fairness metrics on COMPAS datasets including DI, AOD, SPD, EOD, and TI across all models through calculating composite scores. **(A)** Race Before (12, 000 samples). **(B)** Race After (80, 000 samples). **(C)** Sex Before (8, 000 samples). **(D)** Sex After (8, 000 samples).

Figure 7 illustrates an example of navigating the trade-off by computing the composite score across all models. Figures 7A, B depict performance results for the protected attribute Race before reweighting with 12,000 samples and after reweighting with 8,000 samples, respectively. Similarly, Figures 7C, D present results before reweighting with 8,000 samples and after reweighting with 80,000 samples for Race. The findings indicate that, prior to reweighting, RF outperformed other models, whereas DT achieved the highest *Score*_*CS*_ after reweighting, as shown in Figures 7A, B. For the protected attribute Sex, LR exhibited superior performance before reweighting, while DT performed best after reweighting.

**Figure 8:**
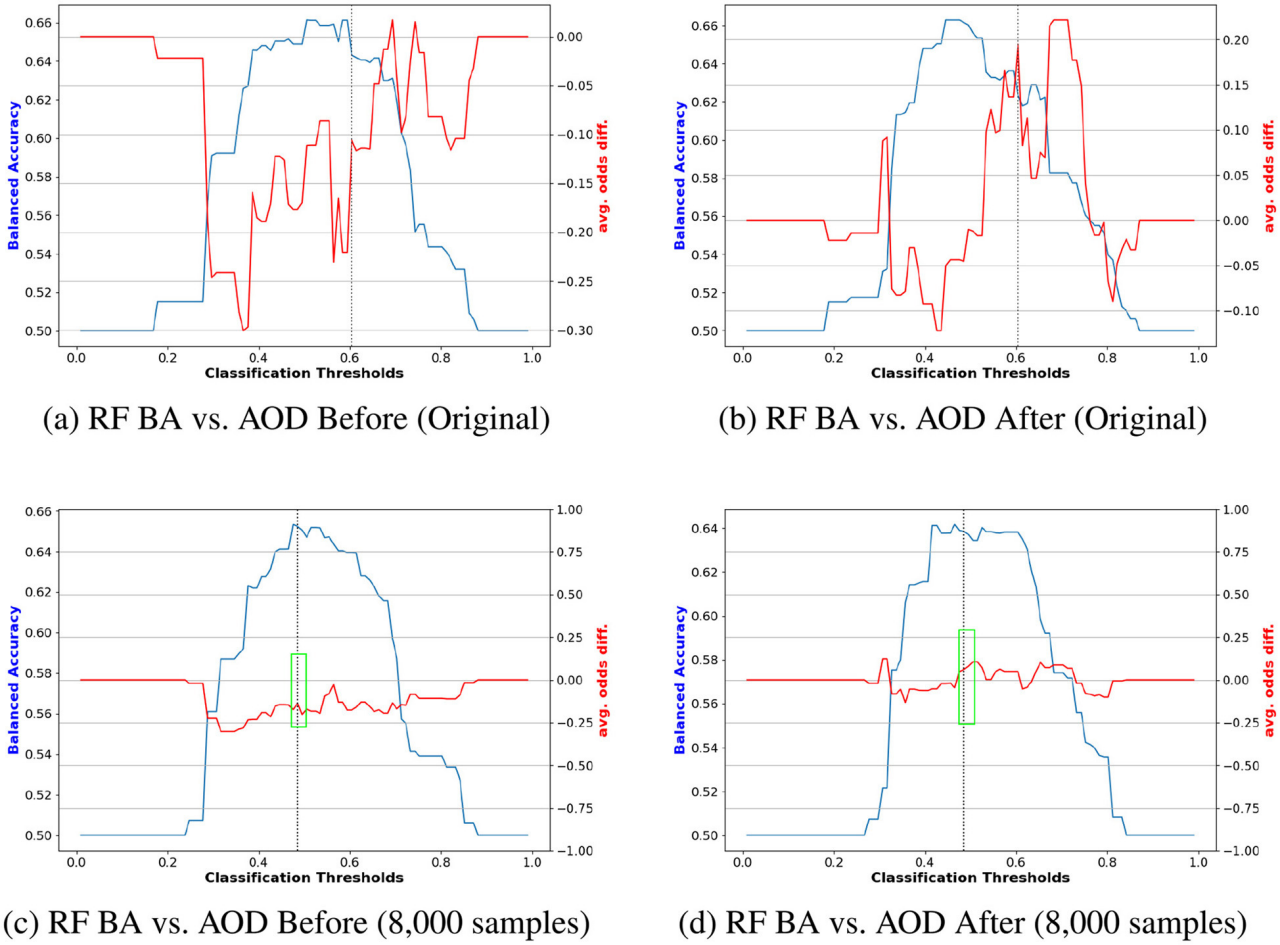
Performance comparison of BA and AOD in augmenting training data with 8, 000 synthetic samples on the COMPAS dataset, considering the protected attribute *Race* for RF. Subfigures **(A)** and **(C)** illustrate the performance comparison between the original training data and the augmented training data (with 8, 000 synthetic samples) before reweighting the samples. In contrast, Subfigures **(B)** and **(D)** present the comparison after reweighting the samples. **(A)** RF BA vs. AOD Before (Original). **(B)** RF BA vs. AOD After (Original). **(C)** RF BA vs. AOD Before (8,000 samples). **(D)** RF BA vs. AOD After (8,000 samples).

**Figure 9:**
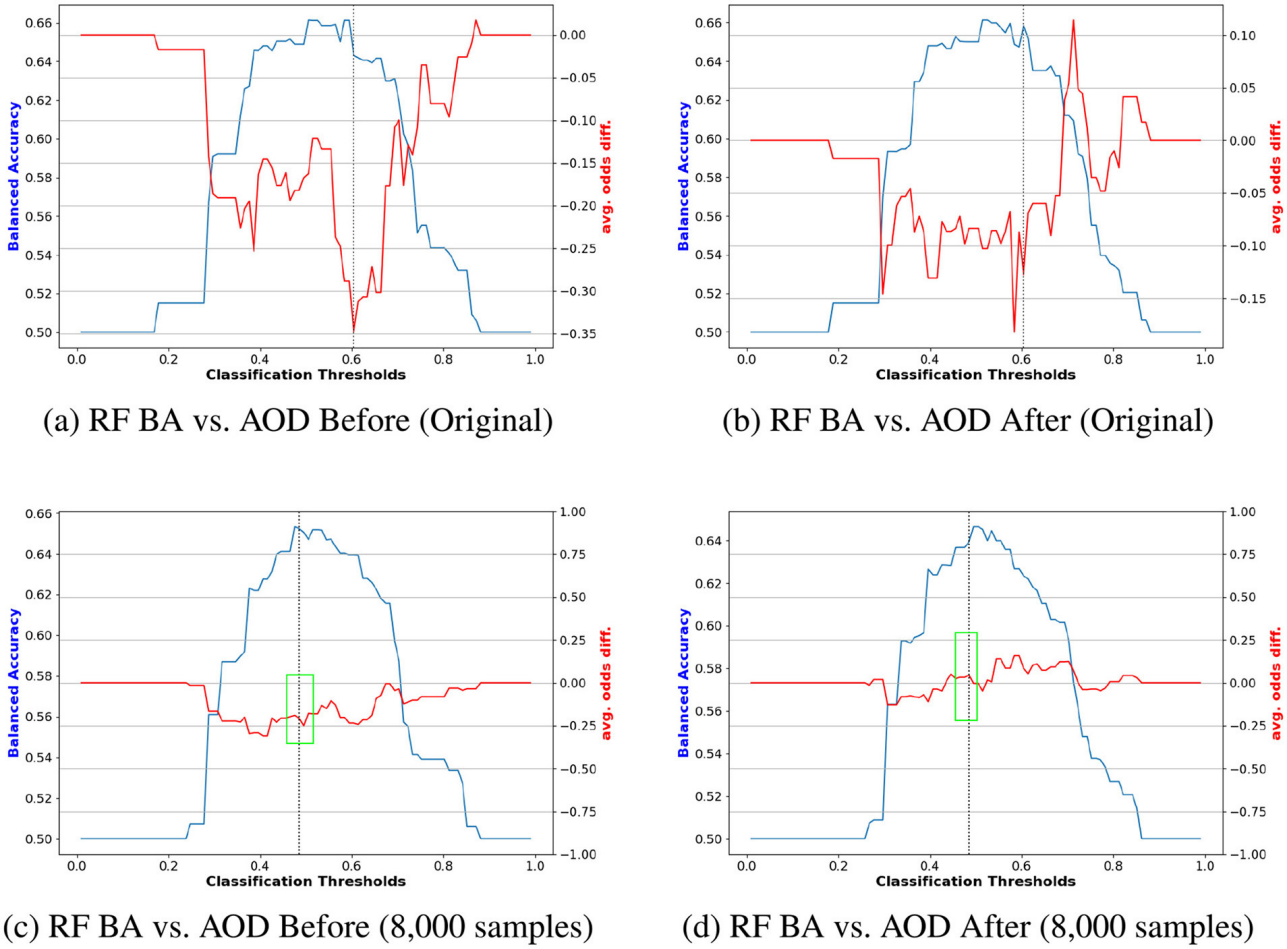
Performance comparison of BA and AOD in augmenting training data with 8, 000 synthetic samples on the COMPAS dataset, considering the protected attribute *Sex* for RF. Subfigures **(A)** and **(C)** illustrate the performance comparison between the original training data and the augmented training data (with 8, 000 synthetic samples) before reweighting the samples. In contrast, Subfigures **(B)** and **(D)** present the comparison after reweighting the samples. **(A)** RF BA vs. AOD Before (Original). **(B)** RF BA vs. AOD After (Original). **(C)** RF BA vs. AOD Before (8,000 samples). **(D)** RF BA vs. AOD After (8,000 samples).

Figures 8, 9 compare the original outputs for RF vs the data augmented outputs for 8, 000 for *race* and *sex*. Reweighting samples further amplifies the benefits of synthetic augmentation by reducing the variability in fairness metric values. Near the optimal trade-off point, highlighted in the green box, the augmented data demonstrates greater stability, as reflected in the smaller fluctuations.

In summary, data augmentation is a valuable approach for improving model fairness in machine learning. However, different models respond uniquely to synthetic data augmentation, underscoring the importance of selecting an appropriate model to achieve the desired balance between fairness and performance.

## 4 Related work

### 4.1 Generative models

Generative models have a rich history in artificial intelligence, starting in the 1950s with the development of Hidden Markov Models (HMMs) (Knill and Young, 1997) and Gaussian Mixture Models (GMMs) (Reynolds et al., 2009), which were used to generate sequential data. However, significant advancements in generative models occurred with the rise of deep learning. In natural language processing (NLP), traditional methods for sentence generation involved learning word distributions using N-gram language models (Bengio et al., 2000) and then searching for the best sequence. To handle longer sentences, recurrent neural networks (RNNs) (Mikolov et al., 2010) were introduced for language modeling tasks, allowing for the modeling of relatively long dependencies, a capability enhanced by Long Short-Term Memory (LSTM) and Gated Recurrent Units (GRU), which use gating mechanisms to control memory during training. These methods can effectively attend to approximately 200 tokens in a sample manner (Khandelwal et al., 2018), marking a substantial improvement over N-gram models. In computer vision (CV), Generative Adversarial Networks (GANs) (Goodfellow et al., 2020) have achieved remarkable results across various applications. Additionally, Variational Autoencoders (VAEs) (Kingma, 2013) and diffusion models (Song and Ermon, 2019) have been developed to provide more fine-grained control over the image generation process, enabling the creation of high-quality images.

### 4.2 Diffusion models

Diffusion models are powerful tools for generating synthetic data. The Denoising Diffusion Probabilistic Model (DDPM) is a type of latent variable model inspired by non-equilibrium thermodynamics, using a Gaussian distribution for data generation (Nichol and Dhariwal, 2021). These models are not only simple to define but also efficient to train, and they can be integrated with non-autoregressive text generation methods to improve text generation quality (Li et al., 2023b). Song et al. (2020) introduced a stochastic differential equation (SDE) that gradually transforms a complex data distribution into a known prior distribution by adding noise, and a reverse-time SDE that reconstructs the data distribution from the prior by gradually removing the noise. The reverse-time SDE relies solely on the time-dependent gradient field of the perturbed data distribution. Vahdat et al. (2021) proposed the Latent Score-based Generative Model (LSGM), a new method that trains Score-based Generative Models (SGMs) in a latent space within the framework of variational autoencoders for image generation.

### 4.3 Reweighting samples for AI fairness

AI fairness has emerged as one of the most critical challenges of the decade (Shaham et al., 2023). Although machine learning models are designed to intelligently avoid errors and biases in decision-making, they can sometimes unintentionally perpetuate bias and discrimination within society. Concerns have been raised about various forms of unfairness in ML, including racial biases in criminal justice, disparities in employment, and biases in loan approvals (Angwin et al., 2022). The entire lifecycle of an ML model, from input data through modeling, evaluation, and feedback, is vulnerable to both external and inherent biases, which can lead to unjust outcomes. Techniques to mitigate bias in ML models are generally divided into three categories: pre-processing, in-processing, and post-processing (Caton and Haas, 2020). Pre-processing recognizes that data itself can introduce bias, with distributions of sensitive or protected variables often being discriminatory or imbalanced. For example, Blow et al. (2024) conducted a systematic study of reweighting samples for traditional ML models, using five models for binary classification on datasets such as Adult Income and COMPAS, and incorporating various protected attributes. Notably, the study leveraged AI Fairness 360 (AIF360), a comprehensive open-source library designed to identify and mitigate bias in machine learning models throughout the AI application lifecycle.

## 5 Conclusion

Understanding the impact of generative modeling is crucial to preventing unintended bias when augmenting training data. This study explores data augmentation via diffusion models, aiming to reduce bias and improve overall performance. It involved evaluating model performance with the generated data added in various increments to the original dataset, and comparing the results to the original outputs using metrics including balanced accuracy and fairness metrics. Experimental results indicated the effectiveness of synthetic data generated by diffusion models for data augmentation. Future work will build on this exploration by incorporating additional datasets and comparing the effects of varying data increments. Additionally, different tools from AI Fairness 360 (AIF360) will be tested to further mitigate bias.

## Data Availability

The original contributions presented in the study are included in the article/supplementary material, further inquiries can be directed to the corresponding author.

## Author disclaimer

The views and conclusions contained in this document are those of the authors and should not be interpreted as representing the official policies, either expressed or implied, of the NSF or the Army Research Office or the U.S. Government. The U.S. Government is authorized to reproduce and distribute reprints for Government purposes notwithstanding any copyright notation herein.
